# Postweaning Exposure to Aflatoxin Results in Impaired Child Growth: A Longitudinal Study in Benin, West Africa

**DOI:** 10.1289/ehp.6954

**Published:** 2004-04-27

**Authors:** Yunyun Gong, Assomption Hounsa, Sharif Egal, Paul C. Turner, Anne E. Sutcliffe, Andrew J. Hall, Kitty Cardwell, Christopher P. Wild

**Affiliations:** ^1^Molecular Epidemiology Unit, Centre for Epidemiology and Biostatistics, Leeds Institute of Genetics Health and Therapeutics, Faculty of Medicine and Health, University of Leeds, Leeds, United Kingdom; ^2^International Institute of Tropical Agriculture, Cotonou, Benin, West Africa; ^3^London School of Hygiene and Tropical Medicine, London, United Kingdom

**Keywords:** aflatoxin, biomarkers, child growth, dietary exposure, longitudinal study, weaning

## Abstract

Aflatoxins are dietary contaminants that are hepatocarcinogenic and immunotoxic and cause growth retardation in animals, but there is little evidence concerning the latter two parameters in exposed human populations. Aflatoxin exposure of West African children is known to be high, so we conducted a longitudinal study over an 8-month period in Benin to assess the effects of exposure on growth. Two hundred children 16–37 months of age were recruited from four villages, two with high and two with low aflatoxin exposure (50 children per village). Serum aflatoxin–albumin (AF-alb) adducts, anthropometric parameters, information on food consumption, and various demographic data were measured at recruitment (February) and at two subsequent time points (June and October). Plasma levels of vitamin A and zinc were also measured. AF-alb adducts increased markedly between February and October in three of the four villages, with the largest increases in the villages with higher exposures. Children who were fully weaned at recruitment had higher AF-alb than did those still partially breast-fed (*p* < 0.0001); the major weaning food was a maize-based porridge. There was no association between AF-alb and micronutrient levels, suggesting that aflatoxin exposure was not accompanied by a general nutritional deficiency. There was, however, a strong negative correlation (*p* < 0.0001) between AF-alb and height increase over the 8-month follow-up after adjustment for age, sex, height at recruitment, socioeconomic status, village, and weaning status; the highest quartile of AF-alb was associated with a mean 1.7 cm reduction in growth over 8 months compared with the lowest quartile. This study emphasizes the association between aflatoxin and stunting, although the underlying mechanisms remain unclear. Aflatoxin exposure during the weaning period may be critical in terms of adverse health effects in West African children, and intervention measures to reduce exposure merit investigation.

Aflatoxins are fungal metabolites that contaminate dietary staple foods such as groundnuts and maize in agroecologies where hot, humid climates combine with poor food storage conditions to facilitate fungal growth and toxin production [International Agency for Research on Cancer (IARC) 2002]. Aflatoxins are proven hepatocarcinogens in many animal species. In populations in parts of Africa and Southeast Asia, exposure is associated with an increased risk of hepatocellular carcinoma, particularly in individuals with chronic hepatitis B virus infection (Hall and Wild 1994; IARC 2002; Wild and Turner 2002). In addition to their carcinogenic properties, aflatoxins can cause growth retardation and impairment of immune function in animals (Raisuddin et al. 1993). However, to date there has been little investigation of these latter parameters in exposed human populations. In one study of Gambian children, Turner et al. (2003) found evidence of a reduced level of salivary immunoglobulin A (IgA) in exposed individuals but no effect on antibody titers to pneumococcal and rabies vaccines.

Aflatoxin exposure cannot be measured accurately at the individual level through a combination of questionnaire-based approach and food analysis, primarily because the heterogeneity of toxin distribution within a particular food product makes representative sampling impractical. Exposure biomarkers have been developed to circumvent this problem, including serum aflatoxin–albumin (AF-alb) adducts that reflect recent past exposure (previous 2–3 months) (Wild and Turner 2002). In a cross-sectional study in Benin and Togo, young children showed a consistently high prevalence and level of AF-alb, with detection of the marker in 99% of children [geometric mean (GM), 32.8 pg/mg; 95% confidence interval (CI), 25.3–42.5]. Exposure was significantly related to weaning status in children 1–3 years of age, with mean AF-alb levels approximately 2-fold higher in fully weaned children compared with those receiving a mixture of breast milk and solid foods. Furthermore, the level of AF-alb was strongly associated with growth faltering, particularly stunting (Gong et al. 2002, 2003). Although breast milk may contain aflatoxins (Zarba et al. 1992), these are generally less toxic metabolites (AFM_1_) than are the parent toxins found in the diet (AFB_1_, AFG_1_), and they occur at lower levels. Thus, breast-feeding provides a period of relatively low aflatoxin exposure in a population whose primary weaning foods, particularly maize, are at high risk of contamination. Toxin exposure during the postweaning period may be a critical factor in young children in determining the adverse health effects of aflatoxins in terms of growth, immune status, and eventually liver cancer risk.

Our earlier study of aflatoxin in relation to weaning and growth was of cross-sectional design (Gong et al. 2002, 2003). The study reported here is of longitudinal design over 8 months examining these associations with repeat measures of aflatoxin exposure and anthropometry.

## Materials and Methods

### Subject recruitment and survey time.

Fifty children (16–37 months of age) from each of the four villages (Bagbe, Sedje, Djidja, and Dovi-Cogbe) in Benin were recruited into the study in February 2001 and were followed up in June and October 2001. Bagbe and Sedje are located in the coastal savannah (CS), the southernmost zone in the country, and were expected to have lower aflatoxin exposure. Djidja and Dovi-Cogbe are in southern Guinea savannah (SGS), the zone immediately to the north of CS, and were expected to have higher aflatoxin exposure. Rainfall and humidity decrease from south to north (Hell et al. 2000a). The two agroecologic zones each have two maize-growing seasons per year. SGS was the zone with the highest aflatoxin exposure in the country in our previous study (Gong et al. 2002, 2003). Ethical approval was obtained from the Ministry for Health in Benin. The head of household and the mother of the chosen child were informed about the nature of the study and, if they agreed to participate, signed a statement of informed consent.

A questionnaire, administered by trained interviewers to the mothers of children recruited to the study, obtained information on the child, namely, age, sex, food consumption (including frequency of maize and groundnut consumption during 3 days before blood sampling), weaning status, weaning foods, and general health status. Information was also obtained at each of the later two survey points. Additional data were gathered concerning the economic status of the household and the mother. These were used to generate an index of relative socioeconomic status (SES) based upon actual material belongings and potential for income generation. Questionnaires were administered at each of the three survey periods. Only the mother’s SES was used in the analysis because it was considered more relevant to the child’s diet (Gong et al. 2002, 2003). The mother’s mean SES calculated from the February and October surveys was used for the analysis. Data collected at the February survey with regard to personal information (parent’s religion and ethnicity and mother’s education and body mass index) were used in the analysis.

### Aflatoxin exposure assessment.

A 5-mL blood sample was obtained from each child at each survey date. The serum was separated and the samples stored at –20°C in Benin before shipment on dry ice to the University of Leeds for analysis. The levels of AF-alb adduct were determined after albumin extraction, digestion, and enzyme-linked immunosorbent assay (ELISA) as previously described (Chapot and Wild 1991). The detection limit was 3 pg of aflatoxin-lysine equivalents per milligram albumin. Controls included three positive and one negative control analyzed alongside batches of samples. Samples were measured in ELISA in quadruplicate on at least two occasions on separate days.

### Blood micronutrients.

Plasma vitamin A was measured by reverse-phase high-performance liquid chromatography by the method of Thurnham et al. (1988) with the minor modification that hexane was used for extraction instead of heptane. Zinc was measured by atomic absorption spectroscopy.

### Anthropometry.

Child and mother’s body weight and height were measured at all three survey dates, using accurately calibrated instruments [electronic scales: Soehnle (BCB Ltd., Cardiff, UK), 20 kg maximum weight, accurate to 10 g; height measurement: Schorr (Olney, Maryland, USA)]. Field workers, trained to maximize repeatability, made all height and weight measurements. Height for age *Z*-score (HAZ), weight for age *Z*-score (WAZ), and weight for height *Z*-score (WHZ) were calculated at the end of the study (October) as described previously (Gong et al. 2002, 2003), according to the World Health Organization reference population (WHO 1986).

### Statistical analysis.

The AF-alb adduct data were not normally distributed and were natural log transformed for statistical analysis. The mean AF-alb level from all three surveys for a given individual was calculated and used as a measure of aflatoxin exposure in some of the analyses. Growth velocity was calculated either as the height difference between two survey points or the difference over the whole 8-month period. The difference between means was tested by *t*-test or analysis of variance (ANOVA). Significant variables of age, village, and mother’s SES were entered into multivariable models to analyze effects of weaning status on AF-alb level and AF-alb on growth velocity. All the analyses were performed using Stata version 8.0 software (StataCorp., College Station, TX, USA). GMs for AF-alb with 95% CIs are reported in the tables and text unless otherwise stated.

## Results

Demographic data for the 200 children at recruitment (February) are presented by village in Table 1. There were no significant differences in age and sex distribution by village. The majority religion was Christian in three of the four villages, with Voodoo being the most common in Djidja. Dovi-Cogbe had the lowest mean measure of mother’s SES, whereas Djidja had the highest. In terms of the major dietary sources of aflatoxin during the period of the study, most of the children (> 80%) in all four villages had consumed maize (including in weaning foods) daily in the 3 days before recruitment in February, and this pattern was maintained in the latter two surveys. In contrast to the almost uniform consumption of maize, the frequencies of groundnut consumption in February in the four villages did differ significantly (*p* < 0.0001). Groundnut consumption was more common at this time in Djidja and Dovi-Cogbe than in the other villages (Table 1). The same general pattern was also found at the two later survey points, except for somewhat increased groundnut consumption in Bagbe in June (data not shown).

### AF-alb levels.

AF-alb was detected in almost every individual at all time points, with a prevalence of 98, 99.5, and 100% in February, June, and October respectively. At the individual level, AF-alb showed a highly significant positive correlation between the three survey points (*r* = 0.6253 for February vs. June, 0.5624 for February vs. October, and 0.5398 for June vs. October; *p* < 0.0001 in all cases), suggesting that individuals track over time in terms of their exposure level, although this was predominantly a feature of the higher exposure villages (data not shown). There was no association between AF-alb adduct levels and sex of the child or mother’s SES, body mass index, or level of education. Although there were some differences in adduct level in relation to religious group, these differences were not significant in multivariable analysis (data not shown). Mother’s SES was included in the multivariable analysis because of the relatively strong rationale behind this parameter affecting the child’s diet and hence aflatoxin exposure and, more generally, as a way of controlling for unmeasured dietary confounding. In addition, plasma vitamin A was measured in February and June and zinc in June to assess whether dietary deficiency in these nutrients has a confounding effect on the association between aflatoxin exposure and growth. However, neither nutrient was correlated with AF-alb level (data not shown).

More frequent groundnut consumption was correlated with higher AF-alb in a univariate analysis (Spearman correlation, *p* < 0.0001). However, groundnut consumption did not make a significant contribution to AF-alb level after adjusting for age, weaning status, village, and SES (*p* = 0.256). There was no significant correlation between maize consumption and AF-alb, which is probably explained by the relatively uniform consumption frequency.

**Figure 1:**
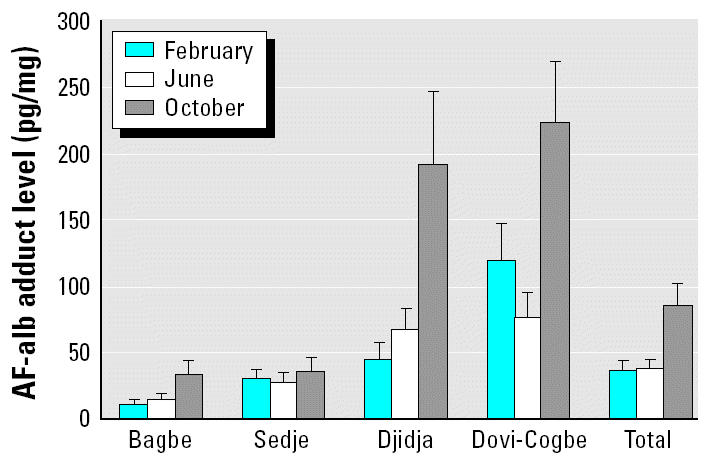
AF-alb adduct GM level across the four villages at the three survey time points. The “Total” bars show the adduct level of all the villages at three survey points, with the overall adduct level at the October survey being significantly higher than the other two survey points (*p* < 0.01). Djidja and Dovi-Cogbe had significantly higher AF-alb than did Bagbe and Sedje at both the October and June survey points (*p* < 0.01 for both). However, at the February survey, the AF-alb adduct levels are different across all the villages (*p* < 0.01).

The AF-alb levels among the four villages showed a similar pattern to that predicted from earlier work, in that Dovi-Cogbe and Djidja had the highest exposures. Longitudinally, AF-alb levels did not differ between February and June (GMs across all four villages, 37.4 vs. 38.7 pg/mg, respectively) but were markedly higher in October (GM, 86.8 pg/mg, *p* < 0.0001) compared with both February and June. This pattern was in general observed for each of the villages individually, although the increase was particularly marked in the two higher-exposure villages, whereas in Sedje there was little variation in AF-alb over the 8-month period (Figure 1).

Village was one of the strongest determinants of AF-alb in the present study together with the time of sampling. To analyze the contribution of both the village and the timing of sampling to the AF-alb, a repeated ANOVA model was used. This analysis showed that village made the major contribution to AF-alb (*F* = 89.7, *p* < 0.0001) followed by the survey time point (*F* = 46.8, *p* < 0.0001).

### Weaning food and weaning practice.

In addition to the data on foods consumed by the children during this 8-month study, we also obtained information concerning the introduction of weaning foods for each child. Because when the children entered the study they were 16–37 months of age, these data were retrospective for most of them. None of the children were receiving exclusively breast milk at the time of the longitudinal study.

The mean age at which children were fully weaned was 22 months, with the youngest recorded age for complete weaning being 9 months. By 3 years of age, all but eight children were completely weaned. In terms of weaning foods, 95% of the children were given a maize-based porridge, although in Bagbe this maize-based porridge was less frequently consumed (only 64%) than in the other three villages, with millet and sorghum used as an alternative. The porridge was introduced quite early in life, with 25% of the children starting from 3 months of age and almost all children (96%) eating this food to some degree by 7 months.

In addition to the porridge or other specified weaning foods, different types of family foods are introduced to the child’s diet between 5 and 12 months of age. Specifically, at 5 months only 6% of children were reported to be receiving family foods additional to the specific weaning foods, whereas by 12 months of age 90% of the children were consuming such foods. The data in Table 1 for maize and peanut consumption refer to all children, both partially and fully weaned. Patterns of weaning across villages were generally similar, although Bagbe had a lower frequency of weaned children at recruitment (Table 1; 44%) even though the age range did not differ from the other villages.

### Weaning and AF-alb.

We examined whether increases in AF-alb with age can be explained by the change in weaning status. As expected in a cohort of this age group, the percentage of fully weaned children increased over the three survey dates from 64% (February) to 79% (June) to 96% (October). When examining the relationship between age and AF-alb, there was a strong positive correlation at recruitment (February), but this became progressively less significant over time and was no longer significant at the end of the 8-month follow-up (*p* = 0.001, 0.033, and > 0.05 for the February, June, and October time points, respectively).

**Figure 2:**
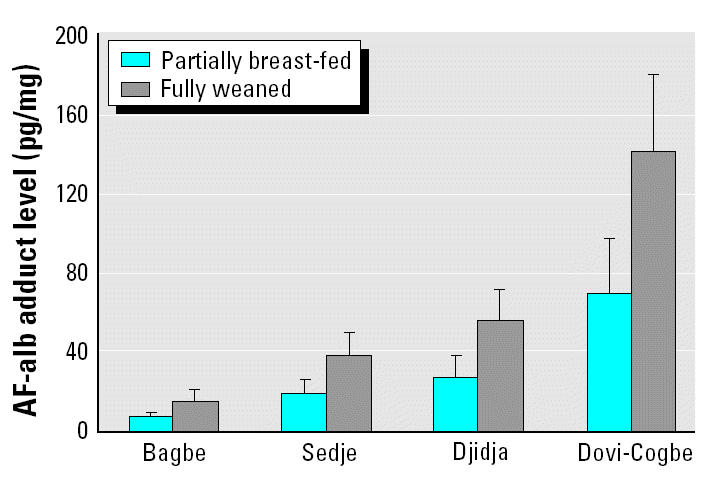
Adjusted mean AF-alb level in weaned and partially weaned children. Data used in this figure are from the February survey point, and the mean AF-alb levels (95% CI) are adjusted for age and SES. The differences between partially breast-fed and fully weaned children are significant in ANOVA, *p* = 0.0001.

To separate the effect of age on AF-alb from that of weaning status, we dichotomized the children in to fully weaned or partially breast-fed groups and examined the age effect in these two separate groups. In this analysis, we found no correlation between age and AF-alb within either group alone (data not shown). Given the small numbers of children still partially breast-fed at the later two survey points, we were able to conduct this analysis only with the February data. When considering the fully weaned group of children compared with those partially breast-fed from all villages in February, we found 2.7-fold higher GM AF-alb level in the former group (53.5 vs. 19.5 pg/mg). The mean AF-alb adduct levels at recruitment in fully weaned and partially breast-fed groups after adjustment for age and SES are shown in Figure 2, revealing a higher mean AF-alb in fully weaned children in each of the villages, even when absolute levels of exposure are significantly different.

To take advantage of the information on change in weaning status over time in individual children, we further categorized the children into four groups. Two groups were partially breast-fed at recruitment (February) but were fully weaned by June or October, respectively, one small group of children (*n* = 7) were partially breast-fed at recruitment and remained so throughout the study, and one group were fully weaned throughout the whole study period. In this analysis (adjusted for age at recruitment, village, and SES), we expressed the results for each child as a ratio of the AF-alb level in October compared with February. The increase in AF-alb was significantly different between the four groups over the 8-month period (*F* = 4.50, *p* = 0.0046; Table 2); however, among the four groups, the greatest increase occurred in children who were fully weaned between February and June and, to a lesser extent, in those fully weaned between June and October. This is further evidence that the change from partial breast-feeding to fully weaned is associated with an increase in aflatoxin exposure.

### Growth and AF-alb.

When AF-alb levels, either in February or the mean level from the three survey points, were analyzed by quartiles, there was a significant inverse correlation with HAZ and WHZ score but not WAZ score. After adjustment for age, sex, height, weaning status (all data from the February point), SES, and village, a significant correlation remained between HAZ and both measures of AF-alb (*p* = 0.009 for February AF-alb, *p* < 0.0001 for mean AF-alb over three survey points), but there was no significant correlation between WHZ and AF-alb.

Height and weight were measured at each of the survey dates. The increase in height and weight between the first and last time point (8 months apart) was calculated and compared with mean AF-alb (represented as quartiles) for each individual over the three survey points or the level at recruitment (February). There was a significant inverse association between mean AF-alb at the three survey points and increase in height but not weight (data not shown) from February to October (Table 3). This association remains highly significant after adjustment for age, height, weaning status (all at recruitment), SES, and village (*p* < 0.0001). The retardation in height increase was 1.7 cm over the 8-month period between the highest and lowest quartiles of aflatoxin exposure (Table 3). In addition, when AF-alb at entry into the study was used as the measure of exposure, the results were quite similar to those found when exposure was integrated over the whole period (Table 3; *p* = 0.003).

## Discussion

The present study confirmed that children in Benin have exceptionally high aflatoxin exposure, with some individual levels of AF-alb (> 1,100 pg aflatoxin-lysine equivalents per milligram albumin) being higher than we have observed in any other population. This biomarker has permitted studies of the health effects of aflatoxin exposure that were previously precluded because of the inability to accurately estimate individual exposure by dietary assessment. Together with previous studies of children in other parts of West Africa using this biomarker, a picture emerges of consistently high levels of exposure in this part of the world (Allen et al. 1992; Turner et al. 2000, 2003). The present data from Benin are consistent with the previous cross-sectional study in Benin and Togo with regard to both high levels and the fact that villages in the SGS (i.e., Djidja and Dovi-Cogbe) were found to have the highest exposure (Gong et al. 2002, 2003). In fact, the AF-alb levels are characterized by marked geographic variations with Dovi-Cogbe, the village with highest levels, having a mean AF-alb 10 times that in Bagbe. Overall, village was the strongest determinant of AF-alb level. Temperature and humidity are two factors that favor growth of *Aspergillus* species and production of the associated aflatoxins as secondary metabolites, and these will vary geographically. Harvest and storage practices also differ from village to village, and these will influence the susceptibility of crops to fungal infestation and toxin production (Hell et al. 2000a).

Seasonal changes in aflatoxin exposure have been reported in previous work, presumably as a result of toxin accumulation during storage under hot, humid conditions, often complicated by insect infestation (Hell et al. 2000a; Turner et al. 2000; Wild and Hall 2000). In the present study, only minor changes in AF-alb were observed between February and June, but there was a substantial increase between June and October in all but one village (Sedje). These dynamics are difficult to relate directly to any specific factor because of variations in annual climatic conditions, the fact that there are two maize harvests per annum, and that maize can be stored for > 1 year. Consequently, the variations in toxin level are more complex than they are for groundnuts, a crop that tends to be eaten within the year following harvest. The AF-alb level will also be influenced by the frequency, quality, and quantity of maize and groundnut consumption throughout the year due to availability of these and alternative food sources.

The observations from this longitudinal study confirmed our earlier report (Gong et al. 2002, 2003) that weaning onto family foods represents a period of increasing aflatoxin exposure. Although age was significantly correlated with AF-alb at recruitment, this association became weaker over the study period. In further analysis, it was apparent that weaning status was the underlying cause of this observation, for the following reasons. First, the correlation at the February survey between age and AF-alb level disappeared when the correlation was considered separately in children categorized as fully weaned or partially breast-fed. Second, when grouping the children according to their change in weaning status over time, we found that it was the change from partial breast-feeding to complete weaning that was correlated with the largest increase in AF-alb. Nevertheless, it is noteworthy that even in those children who continued to receive some breast milk throughout the follow-up, there was a modest increase in AF-alb, possibly reflecting the increasing proportion of total food consumption coming from the weaning and family foods as the child becomes older.

The most likely source of aflatoxin exposure during the weaning period in this population is maize. The main source of aflatoxins will vary by region, and in other parts of West Africa groundnuts are the major contributor to exposure (Turner et al. 2002); in the present study the precision of our dietary analysis does not permit us to completely exclude groundnuts as a contributing factor to aflatoxin exposure, but groundnuts are eaten less frequently and in smaller amounts than is maize. Maize is one of the main dietary staples frequently contaminated with aflatoxins worldwide, including in West Africa (Hell et al. 2000a, 2000b; Jelinek et al. 1989; Setamou et al. 1997). Levels of aflatoxins in maize in Benin have previously been reported to range up to 532 ppb (Hell et al. 2003). Maize-based porridge was found to be the principle weaning food in all four villages, and AF-alb levels increased when this food replaced breast milk, probably because of the lower toxin levels in milk compared with foods. The fact that maize-based porridge as a weaning food is consumed less frequently in Bagbe, the village with the lowest AF-alb level, is consistent with this interpretation that the maize porridge is a major source of aflatoxin. In Bagbe alternative weaning foods were sorghum and millet, and the prevalence of fully weaned children was somewhat lower than in the other villages. Estimated carryover of aflatoxin from dietary intake to milk in animals is around 1%, and similar estimates were made from studies measuring intakes versus excretion in individual women in The Gambia (Zarba et al. 1992). An alternative hypothesis for the increase in AF-alb after weaning is that breast milk could have an effect on the intestinal absorption of aflatoxin or on its metabolism to reactive metabolites once ingested. However, this hypothesis is not supported by any experimental data so far to our knowledge.

Fetal and early childhood environment is considered critical for growth and disease risk in later life (Terry and Susser 2001). Aflatoxin has been shown to cause both immune suppression and growth impairment in animals (Hall and Wild 1994; Raisuddin et al. 1993). Exposure has been linked to kwashiorkor, a severe protein-energy–deficient disease in African children (Hendrickse et al. 1982); however, this association awaits confirmation in appropriately designed epidemiologic studies (Hall and Wild 1994). In a previous cross-sectional study in Benin and Togo, we found an inverse association between HAZ score and AF-alb adduct level in 480 children 1–5 years of age (Gong et al. 2002). Comparatively, growth velocity is more persuasive than a cross-sectional measure in clarifying the growth impairment associated with aflatoxin. In this longitudinal study, the reduction in height increase was significantly correlated both with higher AF-alb level at recruitment and with high mean AF-alb level over the three time points studied. This association was present after adjusting for age, weaning status, height at recruitment, SES, and village. Categorizing the children into quartiles for mean AF-alb over the three time points in the study, there is a mean 1.7-cm reduction in height gain in the highest versus lowest quartile of exposure over just an 8-month period. This corresponded to a difference in GM of 160.2 pg/mg (174.2–14.0 pg/mg) in AF-alb over the 8-month period between the lowest and highest quartiles of exposure. It should be noted that all the levels of aflatoxin exposure in this study are high and chronic in nature compared with developed countries. If the effects on growth were compared with children infrequently exposed to negligible toxin levels, the observations may appear even more striking.

The strong association between aflatoxin exposure and impaired growth may have significant effects on other aspects of child health, such as immunity and susceptibility to infectious diseases. Nevertheless, the underlying biology to explain the effect of aflatoxin on growth is not understood and is important to investigate. Recently, we reported a reduction in salivary IgA in Gambian children exposed to aflatoxin (Turner et al. 2003). If aflatoxins can alter mucosal barriers and affect resistance to intestinal infections, for example, then this would provide one mechanism for the observations we have made on growth impairment. It is also recognized that mycotoxins occur commonly as mixtures; most notably for the present study, aflatoxins would be expected to co-occur with fumonisins in maize (IARC 2002), and the role of possible interactions between these co-contaminants in the underlying mechanisms of growth impairment is of interest. It might be argued that AF-alb is a surrogate marker for food of poor nutritional quality and that reduced dietary intakes of nutrients are the underlying cause of the association between AF-alb and impaired growth. Evidence that this is not the case comes from the fact that blood micronutrient levels (vitamin A and zinc) were not correlated with AF-alb levels in this study. However, we did not have a measure of consumption of other dietary components or of total energy intake. In fact, to fully distinguish the effects of the toxin from other confounding factors in the diet would require a randomized intervention study where the impact of lowering aflatoxin exposure on child immunity, growth, and disease susceptibility can be assessed. This would also permit a better understanding of the relative contribution of aflatoxin to growth impairment in relation to other important determinants in these communities. Given the potential adverse health effects on West African children of this ubiquitous dietary toxin, it is important to evaluate intervention strategies appropriate to these populations (Wild and Hall 2000).

## Figures and Tables

**Table 1:** Descriptive data at the time of recruitment (February) for each of the four villages.

	Village			
Characteristic	Bagbe	Sedje	Djidja	Dovi-Cogbe
AF-alb (pg/mg)*^a^*	11.8 (9.2–15.2)*	31.1 (25.4–38.0)**	45.9 (35.7–59.0)^#^	119.3 (96.2–148.1)^#^,*
Age (months)*^a^*	25 (23.6–26.4)	26 (24.5–28.2)	27 (25.7–29.0)	27 (25.5–28.7)
Sex (male:female)*^b^*	22:28	29:21	26:24	25:25
Religion (C:I:V)*^b^*	41:1:4*	37:1:9*	13:0:30**	40:0:7*
Weaned (%)	44*	66**	72**	74**
Maize consumption^b^,^c^	1:1:3:45	0:0:4:46	0:0:3:47	0:1:5:44
Groundnut consumption^b^,^c^	32:10:7:0*	24:12:6:7**	5:10:12:23^#^	10:8:13:19^#^,*
Mother’s SES*^d^*	10.8 (8.0–13.5)*	11.0 (8.7–13.4)*	14.7 (12.0–17.3)**	8.9 (7.0–10.8)*

Abbreviations: C, Christian; I, Islam; V, traditional/Voodoo.

a Mean (95% CI).

b Ratio.

c Maize and groundnut consumption refers to the number of children (both partially and fully weaned) having consumed the commodity on 0, 1, 2, or 3 days of the 3 days before the survey date.

d Median (25–75%).

* Data with different symbols are significantly different.

**Table 2:** Mean ratio (95% CI) of AF-alb in October compared with February with respect to change in weaning status over the study period.

		GM AF-alb (pg/mg)		Ratio*^a^* of AF-alb in October compared to February (95% CI)	
Weaning group*^b^*	No.*^c^*	February	October	Unadjusted	Adjusted*^d^*
Weaned at entry	123	54.4	99.4	1.8 (1.5–2.2)	1.6 (1.2–2.1)
Not weaned	7	8.9	24.0	2.7 (1.2–6.1)	2.1 (0.9–4.9)
Weaned at June	27	26.4	127.4	4.8 (3.2–7.2)**	4.2 (2.7–6.6)
Weaned at October	24	13.0	44.3	3.4 (2.0–5.8)*	2.9 (1.7–5.0)

a The ratio of the AF-alb level in February and that in October was calculated for each child, and then the GM of these ratios was calculated for each group.

b Children who were fully weaned at the start of the study are categorized as “weaned at entry.” Those who remained partially breast-fed throughout the study period, “not weaned”; those who were partially breast-fed in February but were fully weaned by June or October.

c Nineteen of the 200 children had incomplete information on weaning status or missing data on AF-alb.

d ANOVA test, *p* = 0.0046 after adjusted for age at recruitment, village, and SES.

* *p* = 0.068 compared with weaned at entry group;

** *p* < 0.0001 compared with weaned at entry group.

**Table 3:** Height increase in comparison with AF-alb level [mean (95% CI)].

	Mean AF-alb over 8 months height increase (cm)		AF-alb at February height increase (cm)	
Aflatoxin exposure group*^a^*	Unadjusted	Adjusted*^b^*	Unadjusted	Adjusted*^b^*
Lower quartile	4.9 (4.5–5.3)*,*^c^*	5.9 (5.2–6.6)	4.9 (4.5–5.2)*	5.3 (4.6–6.1)
Mid-lower quartile	4.4 (4.1–4.7)**	5.3 (4.8–5.9)	4.4 (4.1–4.7)	5.0 (4.5–5.5)
Mid-upper quartile	4.1 (3.8–4.5)**	4.8 (4.4–5.2)	4.0 (3.7–4.4)**	4.7 (4.3–5.1)
Upper quartile	4.1 (3.8–4.5)**	4.2 (3.9–4.6)	4.2 (3.9–4.5)**	4.3 (4.0–4.7)

a The quartiles for mean AF-alb over 8 months are < 23.3, 23.3–53.0, 53.0–101.5, and > 101.5 pg/mg. The quartiles for AF-alb in February are < 17.1, 17.1–39.6, 39.6–82.3, and > 82.3 pg/mg.

b Data are adjusted for age, height, and weaning status in February (*p* = 0.003) and for mother’s SES and village over the 8 months (*p* < 0.0001).

c *Data with different symbols are significantly different.
